# Editorial: Women in psychiatry 2025: autism

**DOI:** 10.3389/fpsyt.2026.1963459

**Published:** 2026-08-18

**Authors:** Costanza Colombi, Rita Barone

**Affiliations:** 1 Stella Maris Foundation, Calambrone, Italy; 2 University of Pisa, Pisa, Italy; 3 Department of Clinical and Experimental Medicine, University of Catania, Catania, Italy

**Keywords:** autism spectrum disorder, biological mechanisms, lifespan, social mechanism, women

Autism spectrum disorder (ASD) is a heterogeneous neurodevelopmental condition whose conceptualization has been shaped, over decades, by predominantly male clinical samples. The Research Topic “Women in Psychiatry 2025: Autism” brings together 17 contributions that reflect the aims of the Women in Psychiatry series—challenging male-centered models of autism, deepening understanding of biological and social mechanisms, and pointing toward individualized supports across the lifespan. The Research Topic spans genetic and immunological inquiry, diagnostic equity, comorbidity, psychosocial intervention, and the lived experience of autistic people and their families—together advancing a research landscape that is biologically rigorous, clinically nuanced, and attentive to gender equity.

Several contributions confront the long-standing underdiagnosis and misdiagnosis of autistic females. Minutoli et al. argue for the clinical and scientific urgency of defining a female autistic phenotype, contending that camouflaging—strategies to mask autistic traits—delays diagnosis and increases vulnerability to anxiety, depression, and burnout. Peña-Casquero et al. trace the historical roots of the male-centered diagnostic model, reviewing contemporary prevalence data suggesting that sex differences may be smaller than traditionally reported, and proposing pathways toward a more equitable and inclusive framework. Grzeszak and Pisula apply minority stress theory to synthesize evidence from 104 studies, demonstrating that autistic females face intersectional disadvantage—at the crossroads of gender and neurodivergence—that compounds mental health disparities beyond the sum of its parts. Extending the camouflaging lens transdiagnostically, Carpita et al. show that social camouflaging is elevated in women with borderline personality disorder (BPD) and significantly predicts eating disorder symptomatology, positioning camouflaging as a potential bridge between autistic traits and disordered eating.

The Research Topic also advances neurobiological understanding. Blumenthal et al. review maternal autoantibody-related autism (MARA), detailing how autoantibodies reactive to fetal brain proteins may disrupt neurodevelopment at the cellular level. Cirnigliaro et al. identify a *de novo* loss-of-function mutation in ARHGAP32—a Rho GTPase-activating protein—in a girl with idiopathic ASD, reinforcing the role of RhoGAP family proteins in synaptic plasticity and expanding the genetic spectrum of autism. Yet biology alone does not determine outcomes: McKinney et al. demonstrate, particularly in males with Fragile X syndrome, that neighborhood-level social determinants of health—including healthcare access, economic resources, and social cohesion—explain significant variance in IQ and adaptive behavior even after accounting for the underlying protein deficit, with almost no associations observed in females. This finding bridges neurobiology and social context, underscoring that phenotypic heterogeneity reflects the interplay of genes, environment, and opportunity.

Comorbidity emerges as a central clinical challenge. French et al. provide an umbrella review of 134 systematic reviews, mapping adverse outcomes across mental health, physical health, and social and lifestyle domains—from anxiety and suicidality to epilepsy, unemployment, and premature mortality. This cross-domain synthesis frames the urgency of the more focused clinical contributions. Narzisi et al. compare cognitive and behavioral profiles of children with ASD, ADHD, and their comorbid presentation, finding that ASD+ADHD represents a distinct phenotype marked by compounded cognitive impairments and pervasive emotional-behavioral dysregulation. Hellings et al. present three cases of adult autistic females with recurrent suicidality who responded rapidly to ADHD pharmacotherapy, suggesting that untreated ADHD may be an overlooked, potentially treatable contributor to suicidality in this population. Močnik et al. describe the diagnostic complexities of co-occurring BPD and ASD in an adolescent female, illustrating how overlapping symptoms—emotional dysregulation, impulsivity, interpersonal difficulties—demand multidimensional, longitudinal assessment to avoid misattribution.

A strength of this Research Topic is its attention to modifiable factors and evidence-based interventions. Schweizer et al. evaluate a manualized group psychotherapy program for autistic adults in routine clinical care, reporting improvements in social functioning, self-worth, and depressive symptoms among 115 participants. Sarawgi et al. present Regulating Together, a manualized group intervention targeting emotion regulation in autistic youth, illustrating how autism-specific cognitive and sensory profiles can inform intervention design. Physical activity emerges as a promising avenue: Richter et al. show that a structured program improved executive functioning, sensory processing, and sleep quality in preteens with high-functioning ASD, while Huang et al. demonstrate in a large sample of female college students that physical activity is inversely associated with autistic traits, with social anxiety serving as a partial mediator—identifying both as modifiable targets.

Two contributions broaden the lens to lived experience and cultural meaning. Zhou and Ning explore the motherhood dilemmas and coping strategies of Chinese mothers of autistic children, revealing remarkable maternal resilience within a socio-cultural context that intensifies gendered caregiving expectations. Burnos and Kopacz examine religiosity among autistic adults through cognitive and empirical lenses, finding the relationship between mentalizing difficulties and religious belief to be more complex than early theoretical accounts predicted—highlighting the diversity of inner life on the spectrum. Together, these 17 contributions suggest that autism research must move beyond recognition of heterogeneity toward systems of assessment and care capable of responding to it. Advancing equity demands gender-sensitive diagnostic tools, transdiagnostic approaches to comorbidity, biologically informed yet socially aware models, and interventions that respect autistic people and families in their real contexts. This Research Topic demonstrates that equity in autism research is not only a matter of who is studied, but of how the field conceptualizes, measures, and responds to the full diversity of autistic lives.

